# HIV Prevalence Correlates with High-Risk Sexual Behavior in Ethiopia's Regions

**DOI:** 10.1371/journal.pone.0140835

**Published:** 2015-10-23

**Authors:** Chris R. Kenyon, Achilleas Tsoumanis, Ilan Steven Schwartz

**Affiliations:** 1 HIV/STI Unit, Institute of Tropical Medicine, Antwerp, Belgium; 2 Department of Medicine, University of Cape Town, Cape Town, South Africa; 3 Department of Medical Microbiology, Faculty of Health Sciences, College of Medicine, University of Manitoba, Winnipeg, Canada; 4 Department of Epidemiology and Social Medicine, Faculty of Health Sciences, University of Antwerp, Belgium; University of Liverpool, UNITED KINGDOM

## Abstract

**Background:**

HIV prevalence varies between 0.9 and 6.5% in Ethiopia’s eleven regions. Little has been published examining the reasons for this variation.

**Methods:**

We evaluated the relationship between HIV prevalence by region and a range of risk factors in the 2005 and 2011 Ethiopian Demographic Health Surveys. Pearson’s correlation was used to assess the relationship between HIV prevalence and each variable.

**Results:**

There was a strong association between HIV prevalence and three markers of sexual risk: mean lifetime number of partners (men: r = 0.87; P < 0.001; women: r = 0.60; P = 0.05); reporting sex with a non-married, non-cohabiting partner (men: r = 0.92; P < 0.001, women r = 0.93; P < 0.001); and premarital sex. Condom usage and HIV testing were positively associated with HIV prevalence, while the prevalence of circumcision, polygamy, age at sexual debut and male migration were not associated with HIV prevalence.

**Conclusion:**

Variation in sexual behavior may contribute to the large variations in HIV prevalence by region in Ethiopia. Population-level interventions to reduce risky sexual behavior in high HIV incidence regions should be considered.

## Introduction

Do differences in sexual behavior play an important role in determining differences in HIV prevalence noted between populations? Surprisingly, there is little consent on this issue. The authors of the “Four Cities Study”, for example, argued that differences in prevalence of herpes simplex virus 2 infection and circumcision rates—but not sexual behavior—are responsible for differences in HIV prevalence [1]. Other studies have found that HIV prevalence tracks closely with high-risk sexual behavior [2–4]. These latter studies have included ecological level studies that assessed the association between HIV prevalence and putative risk factors in ethnic groups within countries. Ecological studies are a valid and necessary approach to study this question as the prevalence of sexually transmitted infections (STIs) is largely determined by the STI transmissibility of the local sex network [5]. Network connectivity (an ecological property) is in turn a critical determinant of STI transmissibility [6]. To be meaningful, however, these ecological studies need to be done in countries where there is considerable heterogeneity in HIV prevalence between different subpopulations and where sexual networks are sufficiently segregated [7].

Ethiopia meets both these criteria. In 2011, there was a seven-fold difference in HIV prevalence between regions [8]. Although there are over 80 different ethnic groups, these have been grouped into 9 ethnically-based regions [8]. Ethiopia is constitutionally formed by these 9 regions and 2 chartered cities–Addis Ababa and Dire Dawa [8]. In this paper we establish that there is a high degree of sexual network segregation along ethnic lines. We then test if there is an association between HIV prevalence and various sexual behaviors by region.

## Methodology

### Data

We used the 2005 and 2011 Ethiopian Demographic and Health Surveys (EDHS) for this study. These are the only nationally representative surveys that have assessed HIV prevalence in Ethiopia. They received ethical committee clearance for data analyses such as the one performed here. As a result, no specific ethics committee approval was necessary for this study.

The 2005 and 2011 EDHS both utilized a household-based, two-stage stratified sampling approach which, once weighted, provided prevalence estimates that are representative for the countries 11 regions. The first stage selected Enumeration Areas from the 1994 and 2007 National Censuses respectively. In the second stage, representative samples of 14,645 and 17,817 households were selected in 2005 and 2011. In 2005, all women age 15–49 years in these households and all men age 15–59 years in every second household were eligible for interviewing. Of those eligible, 14,070 women (96%) and 6,033 (89%) men completed the interviews. In 2011, all women age 15–49 years and all men age 15–59 years in all selected households were eligible to be interviewed. Of those eligible, 16,515 women (95%) and 14,110 men (89%) completed the interview. Further details of the surveys are provided in Table 1 and references [8,9].

**Table 1:** Selected survey characteristics including HIV prevalence and response rates in 15–49 year old women and 15–59 year old men by region in 2011 Ethiopian Demographic and Health Survey.

Region	N Men	N Women	Age Men-years	Age Women-years	Survey Response Rate–Men (%) ^a^	Survey Response Rate -Women (%) ^a^	HIV testing Response Rate (%) ^b^	HIV Prevalence (%) ^c^		% in the Poorest Wealth Quintile ^d^	Education Attained (%) ^e^		
								2005% 15–49 years (95% CI)	2011% 15–49 years (95% CI)		None	Primary	Secondary or higher
Tigray	1384	1728	30.7	27.7	90.5	97.2	87.0	2.1 (1.2–3.8)	1.8 (1.2–2.5)	22.5	43.0	42.9	14.1
Affar	1000	1291	30.5	27.7	89.5	96.3	84.7	2.9 (0.9–6.1)	1.8 (1.0–3.1)	48.6	64.8	24.6	10.7
Amhara	1965	2087	30.7	28.0	91.0	95.9	84.9	1.7 (0.9–2.6)	1.6 (1.0–2.5)	20.1	55.6	34.7	9.7
Oromiya	2060	2135	30.1	27.4	94.5	97.1	90.1	1.4 (0.8–2.1)	1.0 (0.6–1.4)	14.2	39.6	48.5	11.9
Somali	715	914	30.9	28.1	82.4	92.6	69.1	0.7 (0.2–2.9)	1.1 (0.6–2.3)	40.0	60.7	29.3	10.0
Benishangul-Gumuz	1139	1259	29.8	27.4	91.2	94.9	86.1	0.5 (0.2–1.9)	1.3 (0.6–2.5)	25.9	47.4	41.5	11.1
SNNP	1699	2034	30.8	28.1	92.7	95.3	87.1	0.2 (0.0–0.6)	0.9 (0.6–1.2)	19.4	35.7	52.3	12.0
Gambela	940	1130	29.4	26.1	86.7	92.0	82.1	6.0 (3.1–10.6)	6.5 (3.7–11.2)	20.8	23.3	54.8	21.9
Harari	972	1101	30.4	27.4	84.6	93.8	71.9	3.5 (2.1–5.6)	2.8 (2.0–4.0)	1.4	25.8	38.5	35.7
Addis Ababa	1318	1741	30.0	26.6	79.9	93.1	69.7	4.7 (3.6–6.5)	5.2 (4.2–6.5)	0.2	10.4	40.4	49.2
Dire Dawa	918	1095	31.2	27.4	84.1	93.4	73.7	3.2 (1.6–6.0)	4.0 (2.9–5.5)	5.2	28.4	37.2	34.4

^a^ The overall women’s/men’s response rate is defined as the eligible women’s/men’s response rate x the household response rate (see report for details [8]).

^b^ The HIV testing response rate is defined as the percentage of eligible persons 15–49 years old who participated in HIV testing. Non-responders included those who refused testing, were absent at the time of the survey or there were technical difficulties with blood taking.

^c^ HIV prevalence is HIV prevalence for 15–49 year women and men. This is the only variable in the table that reports data from both the 2005 and 2011 EDHS.

^d^ This variable refers to the percent of the respondents from this region that were calculated to fall in the poorest 20% (quintile) of the nationally derived wealth band. The wealth quintiles were derived from an asset index in the 2011 EDHS (see reference for details [8]).

^e^ Highest education level attained: Primary and Secondary refer to any primary or secondary schooling attained respectively.

### Measures

#### HIV Testing and Prevalence

All survey participants were asked to provide dried blood spot samples for anonymous HIV testing. Respondents did not have access to the test results but were referred to counseling and testing services in the local area. Dried blood samples were collected and subsequently tested for HIV using the Vironostika® HIV Uni-Form II Plus O (Biomerieux). All positive results were retested with the Murex HIV Ag/Ab Combination test. A third test, the HIV 2.2 western blot (DiaSorin), was used to resolve occasions of discordance between the first and second test results. Overall, 86% of all EDHS respondents who were eligible for testing were interviewed and consented to HIV testing. Response rates were higher in rural (92%) than urban (84%) areas and did differ somewhat by region (Table 1). In this paper we use HIV prevalence by ethnic group in the 2011 EDHS as the outcome variable. HIV prevalence by ethnic group is defined as the percentage of all persons 15–49 years old testing HIV positive.

#### Homophily

The Women’s Questionnaire in the EDHS 2011 collected information about the ethnicity of husbands or live-in partners as well as the region of residence. All men lived in the same region as the women, but were not always from the same ethnic group. The degree of ethnic-homophily per ethnic group was defined as the percentage of married women whose husbands or live-in partners were from the same ethnic group. These calculations were limited to ethnic groups that constituted more than 1% of the 6745 individuals in the couple sample. This comprised 5841 individuals.

### Independent variables

Each of following predictor variables were calculated separately for men and women and were limited to those between the ages of 15–49 years for women and 15–59 years for men.

#### High-risk Sex (Sex with a non-married, non-cohabiting partner)

The percentage of respondents who reported sex with a non-marital, non-cohabiting partner in the past 12 months, amongst all respondents who reported sex in the past 12 months. This was calculated separately for those aged 15–24 and 15–59 years.

#### Lifetime sex partners

The mean number of reported lifetime sex partners amongst all 15–49 year old women or 15–59 year old men.

#### Age at first sex

The mean age of reported first sexual intercourse among those who reported ever having had sex.

#### Pre-marital sex

The percentage of all never-married 15–24 year olds respondents who reported having had sex.

#### Condom use at last sex

The percentage of respondents who reported using a condom at last sex, amongst those who have had sex in the past 12 months.

#### Male circumcision

The percentage of men 15–59 years old who reported being circumcised.

#### Polygamy

The percentage of married women who report that their husband has more than one wife or partner at the time of the survey.

#### Migration

The percentage of all men who reported spending more than one month away from home in the past year.

### Statistical analysis

All analyses are ecological in nature and conducted with HIV prevalence by region in EDHS in 2005 or 2011 as the outcome variable. The analyses were conducted using STATA 13.0 (College Station, TX) and were all adjusted to account for the complex sampling strategies of the surveys using the survey (SVY) command. The analyses were stratified by gender. Pearson’s correlation was used to assess the relationship between HIV prevalence and each variable. Histograms were used to depict the distribution of the number of lifetime sexual partners by gender and region. We compared the mean number of lifetime partners in the highest (Gambela) and lowest (SNNP) HIV prevalence regions. To assess if the difference in lifetime partners was driven solely by a difference in those reporting 10 or more partners, we repeated the analyses excluding these individuals. Non-overlap in 95% Confidence Intervals (CI) was interpreted as representing a statistically significant difference between the two samples.

## Results

**Fig 1:**
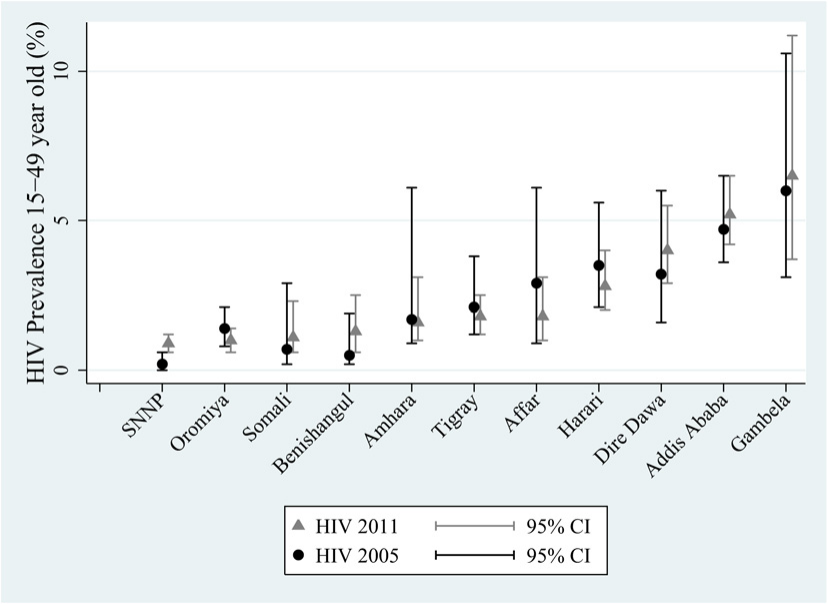
15–49 year old HIV prevalence (%) by region as determined by Ethiopian Demographic Health Surveys 2005 and 2011.

An overview of the numbers, mean ages and other demographic characteristics of men and women by ethnic group that participated in each of the surveys is provided in Table 1. The average age of respondents in the regions ranged from 29.4 to 31.2 years in men and 26.1 to 28.1 years in women. In three of the regions the majority of the population lived in an urban setting and in the remaining 8 regions, 58 to 85% lived in a rural setting. The HIV prevalence by region in 2011 varied between 0.9% (95% CI, 0.6–1.2%) in Southern Nations, Nationalities and Peoples (SNNP) and 6.5% (95% CI, 3.7–11.2%; Table 1) in Gambela. The HIV prevalence estimates produced by the 2005 and 2011 surveys were very similar (Table 1; Fig 1). In 2011, HIV prevalences were 1.6% or below for five regions: Amhara, Benishangul-Gumuz, Oromiya, Somali, SNNP, referred to as the low HIV prevalence regions. HIV prevalences were high (4–6.5%) in three regions: Addis Ababa, Dire Dawa, Gambela; and intermediate (1.8–2.8%) in three regions: Affar, Harari, Tigray. Ethiopia’s two chartered cities (where greater proportions of the populations report higher education outcomes and wealth levels) constituted two of the three high prevalence regions (Table 1). The third high prevalence region, Gambela, is a rural region with high rates of poverty and poor educational attainment.

### Homophiliy

There was a strong tendency for married/cohabiting couples to be from the same ethnic group (see Table 2). The median ethnic-homophily rate for married couples was 92.8% (IQR 89.4–98.3). When restricting the analysis to Addis Ababa the extent of homophilous partnering was reduced (median 70.9% [IQR 67.0–71.2]).

**Table 2:** Homophily by ethnicity. The self-defined ethnicity of married husbands and wives in the 2011 Ethiopian Demographic and Health Survey (Row percentages).

	Husbands Ethnicity														
Wife’s Ethnicity	Affar	Amhara	Berta	Gumuz	Guragie	Hadiya	Kefficho	Nuwer	Oromo	Sidama	Silte	Somalie	Tigrie	Welaita	Total
Affar	344 (99.4)	2	0	0	0	0	0	0	0	0	0	0	0	0	346
Amhara	8	1,408 (87.4)	0	1	22	6	4	0	119	1	2	1	30	9	1,611
Berta	0	0	129 (99.2)	0	0	0	0	0	1	0	0	0	0	0	130
Gumuz	0	0	1	120 (98.4)	0	0	0	0	0	1	0	0	0	0	122
Guragie	0	24	0	0	149 (71.0)	7	0	0	15	0	12	0	3	0	210
Hadiya	0	1	0	0	1	47 (75.8)	0	0	6	0	0	0	0	7	62
Kefficho	0	3	0	0	0	0	92(92.0)	0	4	0	0	0	0	1	100
Nuwer	0	0	0	0	0	0	0	102 (100)	0	0	0	0	0	0	102
Oromo	2	88	5	2	10	0	3	0	1,579 (90.2)	3	2	52	4	1	1,751
Sidama	0	1	0	0	1	0	0	0	0	202 (97.6)	0	1	0	2	207
Silte	0	1	0	0	2	0	0	0	4	0	59 (89.4)	0	0	0	66
Somalie	0	1	0	0	1	0	0	0	24	1	0	341 (92.7)	0	0	368
Tigrie	3	22	0	0	1	0	0	0	3	0	1	2	615 (94.9)	1	648
Welaita	0	2	0	0	1	3	0	0	2	1	0	0	0	119 (93.0)	128
Total	357	1,553	135	123	188	63	99	102	1,757	209	76	397	652	140	5,851

### HIV risk factors

**Fig 2:**
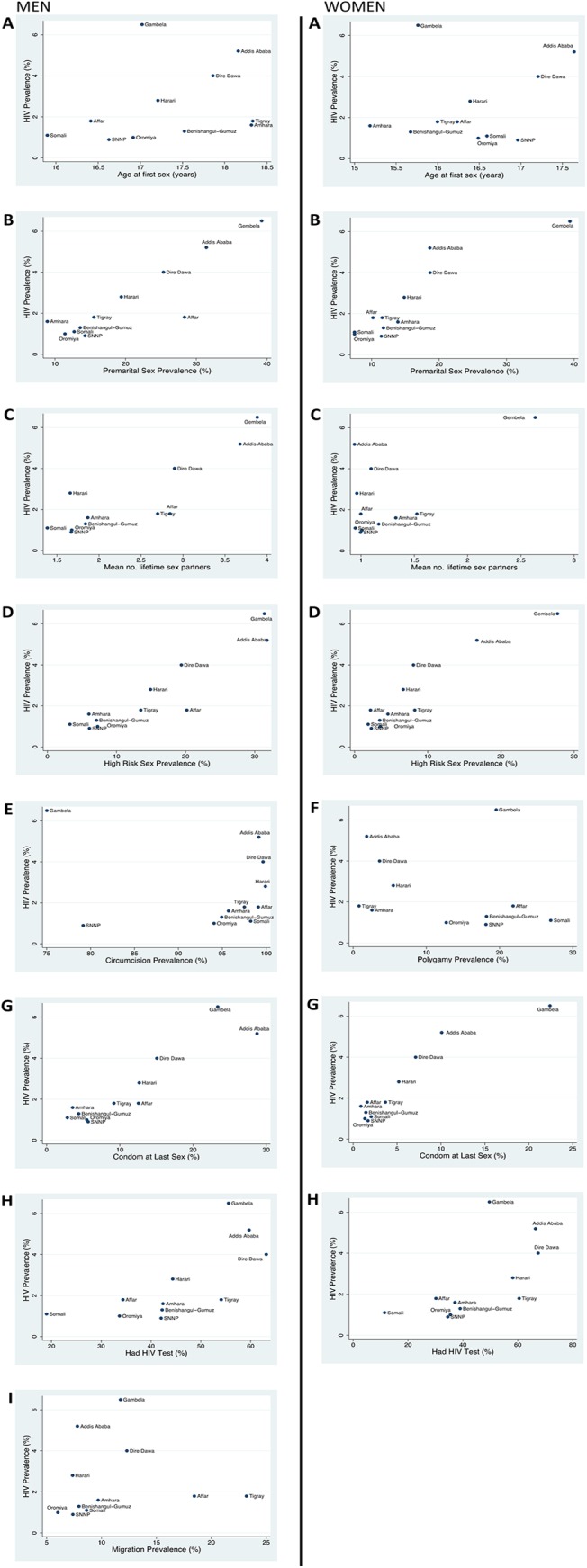
Association between adult HIV prevalence (15–49 years) and various HIV risk factors in 15–59 year old men (left) and 15–49 year old women (right) in the 2011 Ethiopian Demographic and Health Survey. Age of first sex (A), percent reporting pre-marital sex (15–24 year olds; B), mean number of lifetime sexual partners (C), percent reporting sex with a non-married, non-cohabiting partner in prior 12 months (15–59 years; D), the percent of men who reported being circumcised (E), polygamy—percent of married women who report that their husband has other wives (F), condom usage—the percentage who reported using a condom at last sex (G), percent ever tested for HIV (H) and migration–the percent of all men who reported spending more than one month away from home in the past year (I).

The analyses of the correlation between HIV prevalence and various risk factors in both 2005 and 2011 are presented in Table 3. The scatter plots of these relationships for 2011 for each gender are depicted in Fig 2. In the following we describe the results for the correlation analyses conducted on EDHS 2011.

**Table 3:** Pearson’s correlation coefficients for the association between HIV prevalence and various risk factors by region in Ethiopia in 2005 and 2011.

	2011		2005	
	Men	Women	Men	Women
**Age at first sex**				
15–24 years old	0.28	0.23	0.02	0.01
15–59 years old ^a^	0.18	0.33	-0.78 **	0.13
**Pre marital sex (15–24 years old)**	0.89 ***	0.90 ***	0.85 **	0.91 ***
**Mean no. of lifetime sex partners**	0.87 **	0.60	0.89 ***	0.43
**Sex with non-married, non-cohabiting partner**				
15–24 years	0.81 **	0.96 ***	0.76 *	0.57 *
15–59 years ^a^	0.92 ***	0.93 ***	0.90 ***	0.65 *
**Polygamy**	NA	-0.24	NA	-0.05
**Circumcision**	-0.26	NA	-0.46	NA
**Condom usage**	0.92 ***	0.94 ***	0.78 0.005	0.46
**Ever tested for HIV**	0.71 *	0.63 *	0.53	0.47
**Migration**	0.03	NA	0.31	NA

^a^ 15–59 year age category refers to men aged 15–59 years and women aged 15–49 years.

NA—Not Applicable

P-value

* <0.05

** <0.005

*** <0.0005.

### Sexual behavior

#### Age at first sex

Although there was considerable heterogeneity in the reported mean age at first sex for both men and women, there was no association with HIV prevalence. This remained the case when analyses were restricted to those aged 15–24 years.

#### Premarital sex

There was a strong correlation between HIV prevalence and premarital sex for both women and men (r = 0.89; P < 0.001 and r = 0.90; P < 0.001, respectively).

#### Lifetime sex partners

**Fig 3:**
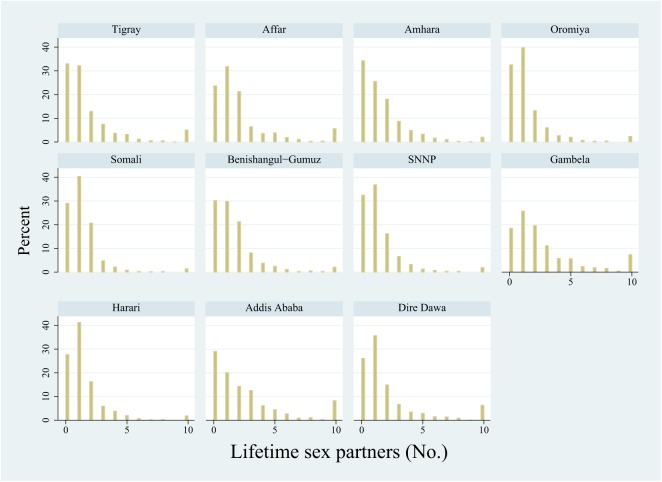
Histograms of the reported number of lifetime sexual partners by region in men aged 15–59 years (EDHS 2011).

There was significant association between HIV prevalence and mean number of lifetime sexual partners for men (r = 0.87; P < 0.001) but not women (r = 0.60; P = 0.05). In the case of men, visual comparison of the histograms from the highest (Gambela) and lowest (SNNP) HIV prevalence regions suggested that the difference in mean number of lifetime partners was not solely driven by a difference in the number with 10 or more partners (Fig 3). There was a significant difference between the mean lifetime partners in men in these two regions (Gambela 4.7, 95% CI 3.7–5.8; SNNP 1.8, 95% CI 1.4–2.2). This difference remained after we excluded those with 10 or more partners (Gambela 2.0, 95% CI 1.8–2.2; SNNP 1.2, 95% CI 1.1–1.3).

#### High-risk sex (Sex with a non-married, non-cohabiting partner)

In both the women and the men, high-risk sex was associated with HIV prevalence (men: r = 0.92; P < 0.001); women r = 0.93; P < 0.001). This relationship remained significant when restricted to those aged 15–24 years (men: r = 0.81; P = 0.003); women: r = 0.96; P < 0.0001).

For premarital sex, high-risk sex and lifetime sex partners (men only) the five low HIV prevalence regions clustered tightly at the lower risk end of scatter plots and the three high HIV prevalence regions clustered together at the high risk end (Fig 2). For each of these three variables, the absolute difference in the value of the risk factor in the high and low prevalence regions was high. For example in 15–59 year old men, the median prevalence of high-risk sex in the five low HIV prevalence regions was 6.0% (95% CI 4.6–7.8%) compared to 31.7% (95% CI 25.9–38.3%) in the three high HIV prevalence regions.

#### Polygamy

There was no statistically significant correlation between the prevalence of polygamy and HIV prevalence (r = -0.24; P = 0.06).

#### Condom usage

Reported condom usage at last sex was low in all ethnic groups but there was a positive association with HIV prevalence (men: r = 0.92; P < 0.001, women: r = 0.94; P < 0.001).

#### Male Circumcision

More than 75% of men in all regions reported having been circumcised. There was no association between circumcision and HIV prevalence (r = -0.26; P = 0.3).

#### Ever tested

Ethnic groups with higher HIV prevalence tended to have a higher percentage of members who reported prior testing for HIV (men: r = 0.71, P = 0.01; women r = 0.63, P = 0.04).

#### Migration

There was no association between migration and HIV prevalence (r = 0.03; P = 0.9).

### 2005 EDHS results

Repeating the analyses using the 2005 EDHS produced very similar results (see Table 3).

## Discussion

HIV has been present in Ethiopia since at least 1984 [10]. Its spread has, however, been far from even in Ethiopia’s 11 regions. A number of studies have examined individual level risk factors for HIV in specific sites in Ethiopia [11–15], or compared changes over time in the whole country [8,9] but there has been little published comparing risk factors across different regions [16]. One exception is a study that evaluated factors associated with HIV infection in 72 000 army recruits from all over the country. The study collected no information on sexual behavior and its major findings were that HIV was associated with urban residence, and in rural areas with a higher level of education and being Orthodox Christian as opposed to Muslim [17].

We found no evidence that risk factors unrelated to sexual behavior were associated with HIV prevalence. Circumcision rates were high throughout Ethiopia and were not associated with HIV prevalence. Condom usage and HIV testing were both positively associated with HIV prevalence, possibly suggesting a greater uptake of these interventions in response to HIV in these areas, as has been found in other countries [4,18]. There was no association between migration and HIV prevalence. Differences in these four risk factors are thus less likely explanations for the observed variations in HIV prevalence.

Our findings are compatible with the thesis that higher risk sexual behaviors play a role in determining the differential spread of HIV in Ethiopia. Three risk factors measuring sexual behavior were associated with HIV: pre-marital sex, number of lifetime sex partners and high-risk sex. These same three risk factors have previously been found to be associated with HIV prevalence by ethnic group in Kenya [2], South Africa [4] and elsewhere [19,20]. Previous reports from DHS data found that at an individual level there was a stepwise increase in HIV prevalence with increasing lifetime sex partners in all 15 countries with available data. This was true for both men and women in all cases [3,8]. This association was also present in the EDHS 2005 and 2011 [8,9]. That this association was present at both individual and population levels increases the likelihood that the association between lifetime sex partners and HIV prevalence is real [21,22]. In our study, although the association was positive for both men and women, it was only statistically significant for the men.

That our findings differed by gender may be due to the fact that DHS-type surveys are particularly prone to underreporting of risky sexual behavior in general and women in particular [23]. A study from Southern Africa found that using Audio Computer Assisted Self-Interviewing compared to standard DHS surveys reduced the male to female ratio of reported lifetime sexual partners from between 2.1 and 4.9 to 1.2 [23]. Other face-to-face interviewing techniques have also been shown to produce more accurate estimates of sexual behavior. A study that compared the estimates of sexual behavior from four African countries derived from DHS versus Population Services International survey methodology found that the DHS surveys produced considerably lower estimates of sensitive sexual behavior such as the prevalence of concurrency [24]. These differences may be related to differences in the length of the surveys, attention paid to conducting the interview in private and the framing of questions [24].

Certain self-reported sexual behaviors are likely to be more accurately captured by the DHS methodology than others [23]. Although this requires further study, sex with partners outside of marriage or cohabitation may be in this category. Reporting on cohabitation with sex partners in the past year is less likely to be susceptible to a response bias than more sensitive information, such as the existence of multiple concurrent partners. The high-risk variable—sex with partners outside of marriage or cohabitation–was constructed based on the insight that relationships with non-cohabiting partners do not offer the same opportunities to monitor sexual exclusivity as cohabiting relationships. As a result of this and other factors, non-cohabiting partnerships may be a marker of increased sexual network connectivity and hence HIV transmission [25]. A large review of individual level data from DHS surveys found that in 15 of the 19 countries with available data, reporting high-risk sex in the past year was strongly associated with HIV infection [3]. At an ecological level, the prevalence of high-risk sex from DHS in Kenya was also found to be associated with HIV prevalence by ethnic group [2]. Further studies are required to test if this relationship obtains in other countries.

This study has a number of limitations. The study is ecological in nature and thus inferences cannot be drawn to an individual level. As already noted, DHS surveys are suboptimal to determine sensitive sexual information. Furthermore, although the response rates for participation in the survey and HIV testing were high, this varied considerably between regions (see Table 1). The data is thus susceptible to a large number of biases such as social desirability, recall and nonresponse biases. In particular, other work has found evidence of culture-specific heterogeneity in answering questionnaires [26,27]. We cannot exclude the possibility that respondents from regions where lower risk sexual behavior was reported were subject to a greater social desirability bias, which could invalidate our findings. The available evidence, however, suggests that only minor differences in sexual behavior exist between those who do and do not answer sexual behavior questionnaires [28]. In addition, the fact that the analyses using the 2005 and 2011 surveys produced such similar results makes the findings more robust. Our analyses are also weakened by the sparse representation of both outcomes (HIV positive/negative) in some of the subsets under investigation. The analyses are all bivariate and thus do not control for the influence of other variables. Finally, we cannot exclude the possibility that the relationships between sexual behavior, region and HIV prevalence are confounded by other unmeasured variables.

## Conclusion

The central finding of this study is of an association between markers of sexual risk and HIV prevalence by region in Ethiopia at both time points for which data is available. We cannot however conclude from this type of study if any particular aspect of sexual behavior plays a dominant role in determining differences in HIV prevalence. Evidence from other sources provides important insights into this question. Studies of high-HIV-prevalence-populations that have managed to reduce HIV incidence–including Uganda, Zimbabwe, Kenya, Thailand and the United States–have all pointed to the importance of reductions in forms of multiple partnering, including partnerships on the side [15,29–31]. Increasing condom usage and newer interventions such as antiretrovirals for treatment-as-prevention [32] and pre-exposure prophylaxis [33] are other important prevention strategies.

This study provides further evidence that differences in sexual behavior play a role in determining the differential spread of HIV. The patterning of behavioural differences suggests that HIV prevention efforts could be enhanced by moving beyond a focus on high-risk individuals to include community-level interventions. The comparison of the distribution of lifetime partners in Gambela and SNNP suggests that the difference between these regions is not just that Gambela has a higher proportion of high-risk individuals but that Gambela’s histogram has been right shifted–in other words, an increase in high-risk behaviour has occurred across the population. Although this requires more research, this finding is suggestive of a difference in norms underpinning this behavior [21,22,34]. Populations with high HIV prevalence in South Africa have been found to have more tolerant norms to the sexual behaviors that underpin HIV spread [34]. If the right-shift in lifetime partners and other variables representing high-risk sexual behaviours in Gambela are similarly driven by populations norms, then HIV prevention efforts that focus only on high-risk individuals would have little effect on the underlying high-risk sexual network. Community lead population level interventions that promote behavior change through social networks may have a more profound effect [15]. The success of these types of interventions in countries such as Uganda has already been noted [15]. A recent randomized controlled trial has further demonstrated the efficacy of this approach [35]. More research is required to better ascertain the constellation of risk factors underpinning the differential HIV prevalence by region in Ethiopia and how these may be addressed.
